# With regard to the original article “A case of primary lung adenocarcinoma mimicking metastatic papillary thyroid carcinoma”

**DOI:** 10.1111/1759-7714.15223

**Published:** 2024-01-18

**Authors:** Yuki Hanamatsu, Yuki Yada, Koyo Shirahashi, Tamotsu Takeuchi

**Affiliations:** ^1^ Department of Pathology and Translational Study Gifu University School of Medicine Gifu Japan; ^2^ One Medicine Center for Innovative Translational Research; Center for One Medicine Innovative Translational Research Gifu University Gifu Japan; ^3^ Department of General Thoracic Surgery Gifu University Hospital Gifu Japan

To the Editor,

In the last issue of *Thoracic Cancer*, Tanaka et al.^1^ reported a unique case of a primary lung adenocarcinoma, which mimicked metastatic papillary thyroid carcinoma (PTC). We read their report with keen interest as we also encountered a similar lung tumor bearing morphological features resembling those of a “PTC‐like lung carcinoma.” Here, we aim to present our case to the readership of *Thoracic Cancer*, seeking to elucidate whether these PTC‐like lung carcinomas are primary lung adenocarcinomas or arise from metastatic PTC conversions. Written informed consent was obtained from the patient in our study.

During follow‐up of a thyroid mass, a man in his 70s was identified as having a single 1.5 cm‐size lung tumor. Histopathological examination of the surgically resected tumor revealed characteristics of an acinar adenocarcinoma with focal papillary growth in the acinar lumen (Figure 1a). A predominant portion of the tumor showed a ground‐glass appearance, along with grooves and pseudoinclusions within the nucleus, which are features typically associated with PTC (Figure 1a,b). We initially believed that the metastatic lung cancer originated from thyroid PTC; however, no cytological evidence for PTC was obtained via needle aspiration analysis of the thyroid mass. Subsequent immunohistochemical studies revealed that the cancer cells displayed negligible immunoreactivity to specific antibodies against PAX8 or thyroglobulin (Figure 1c,d, respectively). Instead, the cancer cells exhibited napsin A immunoreactivity (Figure 1e). Given the robust TTF‐1 immunoreactivity observed (Figure 1f), the immunohistochemical profile of the lung tumor cells suggests a phenotype consistent with primary adenocarcinoma rather than metastatic PTC.

**FIGURE 1:**
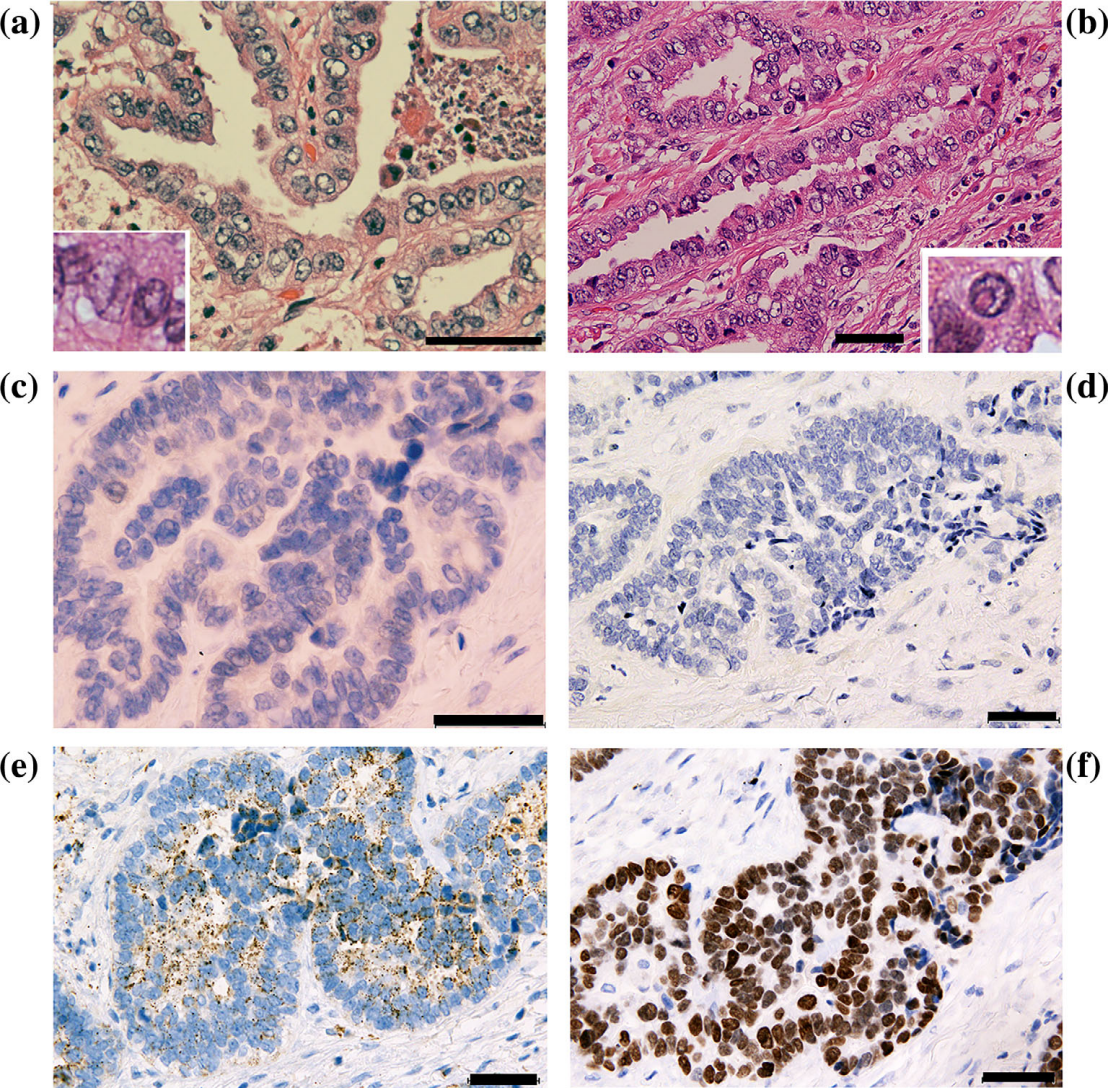
Representative histopathological figures of the lung cancer. (a) Cancer cells demonstrate ground‐glass appearance and grooves in the nucleus. Papillary projection into the tumor lumen was observed. The insert shows typical nuclear grooves. Hematoxylin and eosin (HE) staining. (b) Cancer cells exhibit pseudoinclusions in the nucleus. The insert shows a typical intranuclear pseudoinclusion (HE) staining. (c, d) Representative immunohistochemical staining results. Cancer cells show little or no immunoreactivity to anti‐PAX8 (c) or antithyroglobulin (d) antibodies. In contrast, cancer cells show napsin A (e) and TTF‐1 (f) immunoreactivity. Scale bars represent 50 μm.

To our knowledge, Zhu et al.^2^ were the first to document the surgically resected “PTC‐like primary lung carcinoma”. In the cases documented by Zhu et al. and Tanaka et al. and in our study, cancer cells exhibited characteristic nuclear features of PTC but lacked their immunophenotype. Accordingly, these tumors may be recognized as primary lung adenocarcinomas that mimic metastatic PTC.

In contrast, Takemura et al.^3^ encountered similar lung cancers and concluded that they were metastatic PTC with morphological and immunophenotypic conversions. In their study, patients with PTC and multiple lung tumor nodules exhibited the PTC immunophenotype, except for one rapidly enlarging nodule with a primary lung adenocarcinoma‐like immunophenotype. Notably, the Oncomine Dx target test revealed that both the lung and thyroid tumors harbored an NCOA4::RET fusion, which is extremely rare in primary lung adenocarcinoma.^4^ Accordingly, all tumors were diagnosed as metastases of PTC, whereby the rapidly enlarging nodule was converted to the lung adenocarcinoma‐like morphology.

Immunophenotype conversion occurs in metastatic breast cancer^5^ and is believed to result from the clonal selection of less differentiated receptor‐negative cells following hormonal treatment. Takemura et al. speculated that long‐term iodine‐131 therapy could have contributed to the observed clonal conversion.^3^ In the case reported in the article by Tanaka et al., the patient also received iodine‐131 internal irradiation.^1^ However, in our patient's case, no treatment was administered for the thyroid mass.

We expect that with further accumulation of cases, the existence of PTC‐like primary lung carcinomas may become more apparent.

## AUTHOR CONTRIBUTIONS

All authors read and approved the final manuscript. Conceptualization, Y.H. and T.T.; Methodology, Y.H.; Investigation, Y.H.; Formal analysis, Y.H.; Resources, Y.Y. and K.S.; Writing – original draft, Y.H.; Writing – review and editing, T.T.; Visualization, T.T.; Supervision, T.T.; Funding acquisition, T.T.; Data curation, Y.H.

## CONFLICT OF INTEREST STATEMENT

The authors declare no conflicts of interest.
